# B cell responses to the 2011/12-influenza vaccine in the aged

**DOI:** 10.18632/aging.100541

**Published:** 2013-03-25

**Authors:** Raj K. Kurupati, Senthil Kannan, Xiang, Zhi Xiang, Susan Doyle, Sarah Ratcliffe, Kenneth E. Schmader, Hildegund CJ Ertl

**Affiliations:** ^1^ The Wistar Institute, Philadelphia, PA 19104, USA; ^2^ Biomedical Graduate Group, University of Pennsylvania, Philadelphia, PA 19104, USA; ^3^ GRECC, Durham VA Medical Center and Center for the Study of Aging and Human, Development and Division of Geriatrics, Department of Medicine, Duke University Medical Center, Durham NC 27710, USA; ^4^ Department of Biostatistics and Epidemiology, University of Pennsylvania, Philadelphia, PA 19104, USA

**Keywords:** influenza, B cells, aging, TIV: trivalent influenza vaccine, plasma cells, antibody secreting Cells, CD27, CD38, immunosenescence

## Abstract

Antibody and B cell responses to influenza A viruses were measured over a period of 2 months in 30 aged and 15 middle-aged individuals following vaccination with the 2011/12 trivalent inactivated influenza vaccine by micro-neutralization assays, ELISAs, ELISpot assays and cell surface staining with lineage-defining antibodies followed by multicolor flow cytometry. Both cohorts developed comparable antibody responses to the H3N2 virus of the vaccine while responses to the H1N1 virus were compromised in the aged. ELISpot assays of peripheral blood mononuclear cells (PBMCs) gave comparable results for the two cohorts. Analysis by flow cytometry upon staining of CD19+IgD-CD20- PBMCs with antibodies to CD27 and CD38 showed markedly reduced increases of such cells following vaccination in the aged. Additional analysis of cells from a subset of 10 younger and 10 aged individuals indicated that in the aged a portion of IgG producing cells lose expression of CD27 and reduce expression of CD38.

## INTRODUCTION

Worldwide, the aged constitute an increasingly large and challenging segment of the human population. In the US, approximately 13% of the population is over 65 years of age and this number is projected to increase to 20% of the population by 2050 (US Census Bureau). Diseases and disabilities vary widely among older individuals, a principle of gerontology known as aged heterogeneity [1, 2], which ranges from very fit individuals to unhealthy and functionally impaired individuals. During aging immune responses decline in a process referred to as immunoscenescence. Accordingly, the aged are disproportionally affected by infectious diseases and respond poorly to vaccination.

Immunosenescence affects multiple aspects of both innate [3] and adaptive [4, 5] immunity. The prime correlates of vaccine-induced protection against viral infections however, are B cells, which produce antibodies and show numerous defects upon aging.. B cell lymphopoiesis is reduced with aging, leading to a decline of naïve B cells [6]. Primary B cell responses in the elderly are commonly low and short-lived, resulting in antibodies with low affinity [7]. Formation of germinal centers is decreased [8], antigen transport is impaired and follicular dendritic cells have reduced capacity to form antigen depots [9]. Autoantibodies are more common [10] and the B cell repertoire becomes more restricted [11]. Expression of the E2A-encoded transcription factor E47 is decreased in old splenic B cells, which causes a reduction in the activation-induced cytidine deaminase, needed for class switch recombination and Ig somatic hypermutation [12]. Some of the defects of B cell responses are secondary to an age-related decline of helper functions from CD4^+^ T cells, which show reduced expression of critical co-stimulatory receptors [13,14] that are essential for activation of B cells, germinal center formation and rearrangement and hypermutation of immunoglobulin (Ig) genes.

Influenza is one of the top 10 causes of death in older adults. A trivalent inactivated vaccine for influenza (TIV) consisting of two strains of influenza A and one strain of influenza B virus is approved for use in the elderly, but affords incomplete protection [15,16]. This has been linked in part to poor stimulation of B cells producing virus-neutralizing antibodies. Unexpectedly morbidity and mortality of the H1N1 2009 influenza virus pandemic was by far more common in children and young adults rather than in the aged [17] who experience the highest rates of serious diseases and deaths during seasonal outbreaks. It has been speculated that the aged were in part protected from the pandemic H1N1 virus due to previous exposures to related strains [18]. Other studies showed that the aged paradoxically mounted superior antibody responses to pandemic H1N1 than the young, which were characterized by both broader repertoires and higher avidity [19], again implicating that the aged but not the young mounted recall responses.

To assess responses of the aged to TIV in the post 2009 pandemic phase, we tested B cell responses of 30 aged individuals of or above 65 years of age to the influenza A virus components of the 2011/12 TIV in comparison to a cohort of 15 middle-aged individuals of 30-40 years of age. The objective of the study was to compare antibody and B cell responses of the two cohorts with regard to magnitude and kinetics of responses using three complementary assay systems. As expected most individuals of the middle-aged cohort responded to both influenza A virus strains. Aged individuals more commonly responded to the H1N1 virus than to the H3N2 virus. Interestingly within responders, vaccine-induced neutralizing antibody titers to H3N2 were comparable in magnitude between aged and younger individuals while the aged cohort mounted significantly lower neutralizing antibody titers to the H1N1 virus. At baseline, the aged had significantly higher levels of circulating IgG to both viruses compared to younger individuals. Analyses of peripheral blood mononuclear cells (PBMCs) by ELISpot assays showed no difference in responses between younger and aged individuals suggesting that low antibody responses in the aged related to cell intrinsic defects rather than lack of responding cells. Analysis of CD19^+^ PBMCs by staining for CD27 and CD38 to detect antibody secreting cells (ASCs) by flow cytometry, a method which upon influenza virus vaccination primarily detects vaccine-induced cells, showed only marginal increases in circulating ASC in the aged while younger individuals showed far more pronounced increases. Further analysis suggested that in the aged, ASCs downregulate expression of CD27 and CD38. This is an important finding as loss or reduced expression of these two molecules, which are both involved in crucial signaling pathways, may negatively affect ASC functions.

## RESULTS

### Human Cohorts

The average age of the aged cohort was 74 ranging from 65-87. As shown in Table 1A, 20 individuals were female, 10 were male. The majority [20] was Caucasian, two were African Americans, one was of mixed ethnicity and one was either American Indian or Alaskan Native. Twenty reported influenza vaccination during the previous 5 years, 3 were unsure and 2 reported influenza-like illnesses. Thirteen of those that reported previous vaccinations had been immunized in 2010 when the pandemic H1N1 strain was incorporated into the vaccine. Sixteen individuals between 30-40 years of age with an average age of 36 were enrolled and 15 completed the study. As shown in Table 1B, 9 of the 15 younger individuals were female and 6 were male, 9 were Caucasian, 5 were African American and 1 was of mixed ethnicity. There were no SAEs following vaccination in either age group.

**Table 1a:** 

ID	Age	Gender (F/M)^1^	Ethnicity (C/AA/AI/AN)^2^	Flu History		Ab response^3^		ELISpot response^4^		ASC response^5^		Clinical observations (visit)
				Vaccination	Infection	H1N1	H3N2	H1N1	H3N2	Day 7^7^	Day 14^8^	
222.01	74	F	C	Annually	None	N, Y	Y, Y	n.t.	n.t.	Y	Y	Runny nose (6)
222.02	71	F	C	None	None	Y, Y	N, Y	Y	Y	Y	N	Sore throat (2,6)
222.03	69	F	C	2010	None	N (+), N	N (+), Y	Y	N (+)	N	N	
222.04	76	M	C	None	None	N, Y	Y, N	Y	Y	N (+)	N	URI (6)
222.05	87	F	C	2007/8/9	None	Y, Y	Y (+), Y	Y	Y	Y	N	
222.06	77	F	C	2009	None	Y, Y	Y (+), Y	N	N	Y	Y	Left sides pain (2)
222.07	79	F	C		None	Y (+), N	Y (+), N	Y	Y	N	N	Vertigo (6)
222.08	84	M	C		None	Y, Y	Y, Y	n.t.	Y	Y	Y	Runny nose (6)
222.09	76	F	C	None	None	N, Y	N, Y	N (+)	Y	Y	Y	
222.10	76	F	C	None	None	N, N	Y, Y	Y	N	N	Y	
222.11	73	F	C	3×	None	Y, N	Y (+), Y	Y	Y	N	Y	Hep A & B vaccination (3), URI, cough (6)
222.12	76	M	C	2007	None	Y, N	Y (+), Y	N	Y	Y	N	
222.13	65	M	C	Annually	None	Y, N	Y (+), Y	Y	Y	N (+)	N	
222.14	73	F	C	1×	None	n.t., N	n.t., Y	Y	Y	N (+)	N	Cough (4)
222.15	76	M	C	1×	None	n.t., N	n.t., N	Y	Y	N	N	
222.16	66	F	C	None	None	Y, Y	Y, Y	Y	Y	n.t.	N	Sinusitis (6)
222.17	74	F	AA	Annually	None	Y, N	N (+), N	Y	Y	n.t.	N.	Cough (6)
222.18	74	M	C	2010	None	Y, Y	Y (+), N	Y	Y	N	N	Upset stomach (2), congestion (5), cold symptoms (6)
222.19	67	F	C	None	2009	Y, Y	Y, Y	Y	Y	N	N	Allergies (5)
222.20	67	F	C	2010	None	Y (+), Y	N (+), Y	Y	Y	Y	N	
222.21	73	F	C	Annually	None	Y (+), Y	Y (+), Y	Y	Y	N	N	Allergies (4)
222.22	85	F	C	2007/8/9/10	None	Y (+), Y	Y, Y	Y	Y	Y	N	Fatigue (5)
222.23	71	M	C	None	None	Y, Y	N, Y	Y	Y	N	N	
222.24	70	F	AA	2007/8/9/10	2009	Y (+), Y	N (+), Y	Y	Y	N (+)	N	
222.25	78	F	AI or AN	2007/8/9/10	None	Y (+), N	Y, N	N	N (+)	Y	Y	
222.26	83	M	C	2007/8/9/10	None	N (+), N	N (+), N	Y	N	N	Y	
222.27	71	M	M	2007/8/9/10	1×	Y (+), N	N (+), N	Y (+)	N (+)	N	N	Runny nose (2)
222.28	68	F	C	2009/10	None	N (+), Y	Y (+), Y	Y	Y	Y	N	Injection site redness (2)
222.29	80	F	C	Annually	None	Y (+), N	N (+), Y	Y	Y	N	N	Headache (4)
222.30	71	M	C	?	None	N (+), N	N (+), Y	Y	N	Y	N	

1 F – female, M – male;

2 C – Caucasian, AA – Afro American, M – Mixed;

3 (+)responses by neutralization assays are shown first, responses by ELISA are shown second; (+) indicates baseline titers ≥1:40;

4 (+) indicates baseline spots ≥ 10 per 10^6^ PBMCs;

5 (+) indicates baseline ASC numbers ≥ 1000/10^6^ CD3-CD14- lymphocytes;

6 Numbers in brackets indicates visit when symptoms were reported;

7 Positive ASC response on day 7 reflects an at least 2 fold increase in cell number over baseline;

8 Positive ASC response on day 14 reflects an at least 2 fold increase in ASC numbers compared to day 10.

**Table 1b:** 

ID	Age	Gender (F/M)^1^	Ethnicity (C/AA/M)^2^	Flu History		Ab response^3^		ELISpot response^4^		ASC response^5^		Clinical observations (visit)
				Vaccination	Infection	H1N1	H3N2	H1N1	H3N2	Day 7^7^	Day 14^8^	
111.01	31	F	C	2010	None	N (+),Y	Y (+), Y	n.t.	n.t.	Y (+)	Y	
111.02	40	F	C			Y, Y	Y, Y	n.t.	n.t.	Y (+)	Y	
111.03	37	M	C	None	None	Y (+), Y	Y, Y	n.t.	n.t.	Y (+)	Y	Cold symptoms (2-5)
111.04	31	F	C	None	2008	Y, Y	Y, Y	n.t.	n.t.	Y	Y	Sore throat (5,6)
111.05	37	M	C	None	None	Y, Y	N, Y	n.t.	n.t.	Y (+)	Y	
111.06	30	M	AA		None	Y, Y	Y (+), Y	Y (+)	N (+)	Y	N	Runny nose (6)
111.08	31	F	M	2010/11?	None	Y (+), Y	Y (+), Y	N	N (+)	N (+)	N	
111.09	38	M	AA	None	None	Y, Y	Y, Y	Y(+)	Y (+)	Y (+)	Y	
111.10	39	F	AA	None	None	Y, N	N, N	Y	N (+)	N (+)	N	Sore throat (5)
111.11	39	F	AA		None	Y, Y	Y, Y	Y	N (+)	Y	Y	Runny nose (3-5) Headache (6)
111.12	39	F	C	None	2011	Y (+), Y	Y, Y	Y(+)	N (+)	Y	N	Headache (6)
111.13	30	M	C	1 or 2×	1 or 2× (?)	Y (+), Y	N (+), Y	Y	Y	Y	Y	Stuffy nose (2-4)
111.14	39	F	C	Annually	None	Y, Y	Y, Y	Y	Y	N	N	
111.15	39	F	C	Annually	None	Y, N	Y (+), Y	N	N (+)	N	Y	Runny nose (2,3), Sinus infection (5)
111.16	38	M	AA	None	None	Y, Y	Y, Y	Y	N	N	Y	

1 F – female, M – male;

2 C – Caucasian, AA – Afro American, M – Mixed;

3 (+)responses by neutralization assays are shown first, responses by ELISA are shown second; (+) indicates baseline titers ≥1:40;

4 (+) indicates baseline spots ≥ 10 per 10^6^ PBMCs;

5 (+) indicates baseline ASC numbers ≥ 1000/10^6^ CD3-CD14- lymphocytes;

6 Numbers in brackets indicates visit when symptoms were reported;

7 Positive ASC response on day 7 reflects an at least 2 fold increase in cell number over baseline;

8 Positive ASC response on day 14 reflects an at least 2 fold increase in ASC numbers compared to day 10.

### Vaccine-Induced Antibody Responses

**Figure 1:**
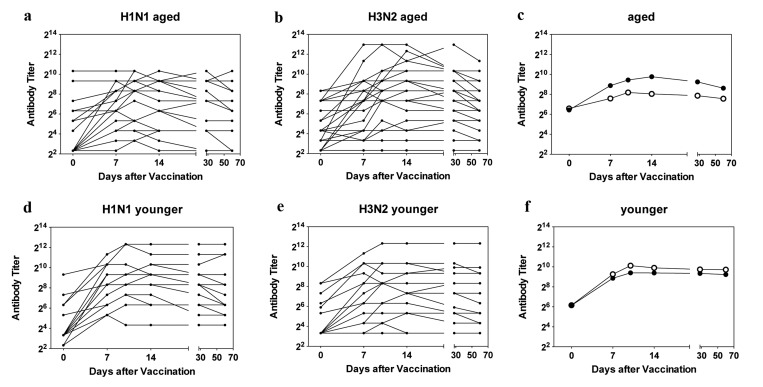
Antibody Titers Titers of H1N1-specific antibodies in individual sera are shown in (**a**) for aged and in (**d**) for younger subjects, titers of H3N2-specific antibodies in individual sera are shown in (**b**) for aged and in (**e**) for younger subjects. **c** shows mean titers of H1N1 (closed circles) and H3N2 (open circles in the aged); **f** shows mean titers in the younger individuals using the same symbols.

Serum samples harvested immediately before and on days 7, 10, 14, 28 and 60 after vaccination were tested for neutralizing antibody titers to H1N1 A/California/04/2009 and H3N2 A/Perth/16/2009 by micro-neutralization assays (Figure 1). A fourfold increase in titers was used to identify responders.

At baseline middle-aged and aged individuals had comparable titers to the two viruses. Percentages of individuals with antibody titers of or above 1:40 to either virus were slightly higher in the aged than in the younger cohorts with 33% of younger vs. 40% of aged being positive for H1N1 virus (p=0.725) and 40% of younger vs. 60% of older individual being positive for H3N2 virus (p=0.466). Aged subjects, who were enrolled towards the end of the study, more commonly had neutralizing antibody titers of or above 1:40 than those that were enrolled earlier. More specifically of the 11 elderly individuals that were first tested after Mid March of 2012, 91% were sero-positive for the H1N1 virus while 72% were positive for the H3N2 virus. In contrast of the 19 aged individuals recruited early only two (i.e., 11%) was positive for H1N1 at the time of vaccination (p<0.001) while 10 (52%) were sero-positive for the H3N2 virus (p=0.490). This bias at baseline was not seen for younger individuals all of whom had been enrolled by the end of February. Upon vaccination 8 aged individuals failed to respond to the H1N1 virus and 10 were unresponsive to the H3N2 virus.

Kinetics of antibody responses differed between the two cohorts. Average titers peaked in the younger cohort by day 10 after vaccination and only marginally declined by the end of the study. In the aged peak titers to H1N1 were also reached by day 10 after vaccination while average peak responses to the H3N2 virus were delayed to day 14 reflecting a significant difference in time to peak response (p = 0.0316). The decline of antibody titers was more pronounced in the aged although this did not reach significance (p = 0.057).

**Figure 2:**
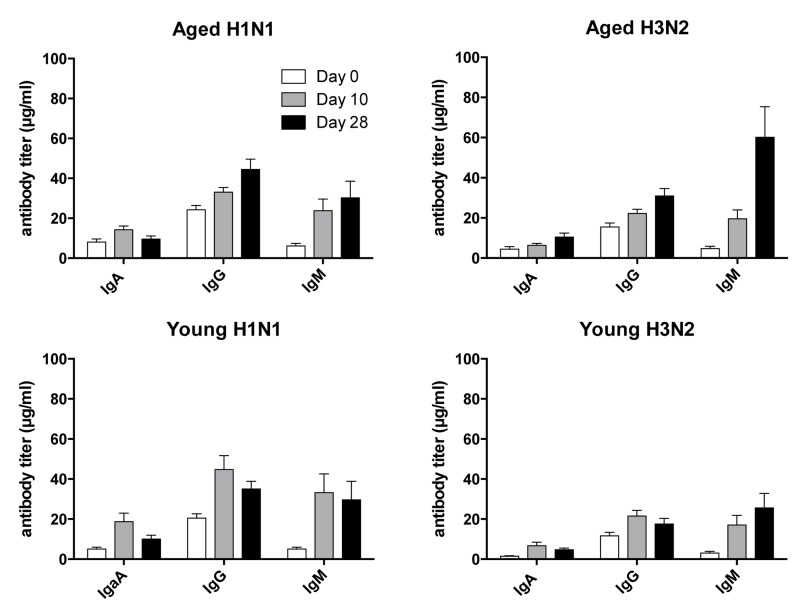
Antibody Isotypes Serum samples collected on days 0, 10 and 28 following vaccination were tested for IgA, IgG and IgM to H1N1 and H3N2 viruses by ELISAs. Graphs show average titers ± SEM.

To test for antibody isotypes, sera collected at baseline and on days 7 and 28 following vaccination were tested by ELISAs on plates coated with H3N2 or H1N1 virus (Figure 2). At baseline, antibody responses were slightly higher in the aged although this only reached significance for H3N2-specific IgA. In both younger individuals IgA and IgM to H3N2 and H1N1 virus significantly increased by day 7 following TIV and this difference remained significant for both cohorts tested on day 28, but for H1N1-specific IgM responses that at this time were no longer significantly higher than at baseline in the aged. IgG responses to H1N1 and H3N2 virus were significantly increased at both time points in the younger individuals while those to H3N2 virus failed to reach significance in the aged on day 28 post vaccination when compared to day 0. Responsiveness by ELISA, defined as a 4 or more fold increase in circulating antibodies of either isotype at either of the two time points differed between the younger and aged individuals. One of the younger individuals failed to show responsiveness to H3N2 virus, while three failed to respond to H1N1; the non-responder to H3N2 virus also failed to respond to H1N1 virus. Non-responsiveness by ELISA was more common in the aged; 3 and 11 individuals failed to show significant increases of antibodies of either isotype to H3N2 and H1N1 virus, respectively on either day 7 or 28. Of the non-responders, the 3 individuals that failed to respond to H3N2 virus also did not develop increased antibodies to H1N1 virus. Aged individuals who did not mount a recall response to H1N1 virus had significantly higher levels of specific IgG at baseline (p<0.05) as compared to responder. The same trend was seen for H3N2 virus but failed to reach significance.

### Vaccine-Induced Circulating Influenza A Virus-Specific ASCs

**Figure 3:**
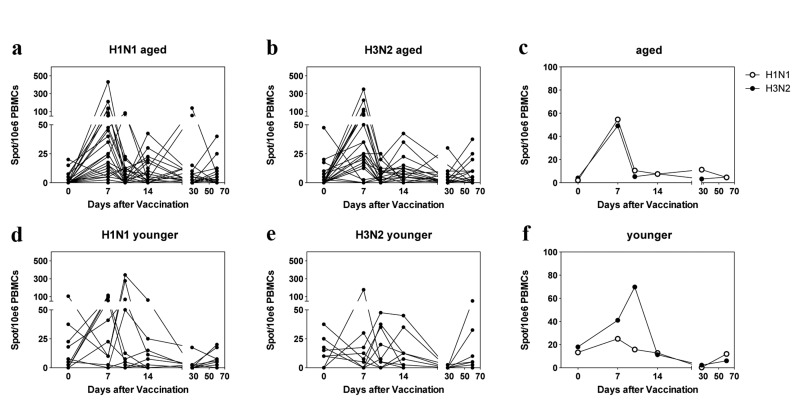
ELISpot Results Spots per 10^6^ live PBMCs are shown for individual samples in (**a**), (**b**) (aged) and (**d**), (**e**) (younger). **c** and **f** show mean results for aged and younger subjects. The graphs are arranged as in Figure 1.

PBMCs from 10 younger and 28 aged individuals were analyzed for vaccine-induced responses to the two influenza A viruses of the vaccine by ELISpot assays (Figure 3). Analyzed samples had been collected just before and then at the 5 above described time points after vaccination. Responders based on this assay had to show at least a 2-fold increase in ASCs and a minimum of ten influenza virus-specific ASCs per 10^6^ PBMCs following vaccination.

At baseline only two versus four aged individuals had ≥10 spots to H1N1 or H3N2 virus, respectively. Three aged individuals failed to respond to the H1N1 vaccine; seven aged subjects did not show an increase in H3N2-specific ASCs. Of the 10 younger individuals, 3 had positive spots for H1N1 virus while 6 were positive for H3N2 virus at baseline. Two younger subjects failed to respond to H1N1 virus; five younger individuals failed to respond to H3N2 virus. The kinetics of responses in the aged showed a sharp peak on day 7 to both viruses, which was followed in some individuals by additional smaller peaks on days 14 or 28. Younger individuals showed peaks either on days 7 or 10. There was no significant difference in magnitude or time to peak responses between the two cohorts.

### Circulating B cell populations

**Figure 4:**
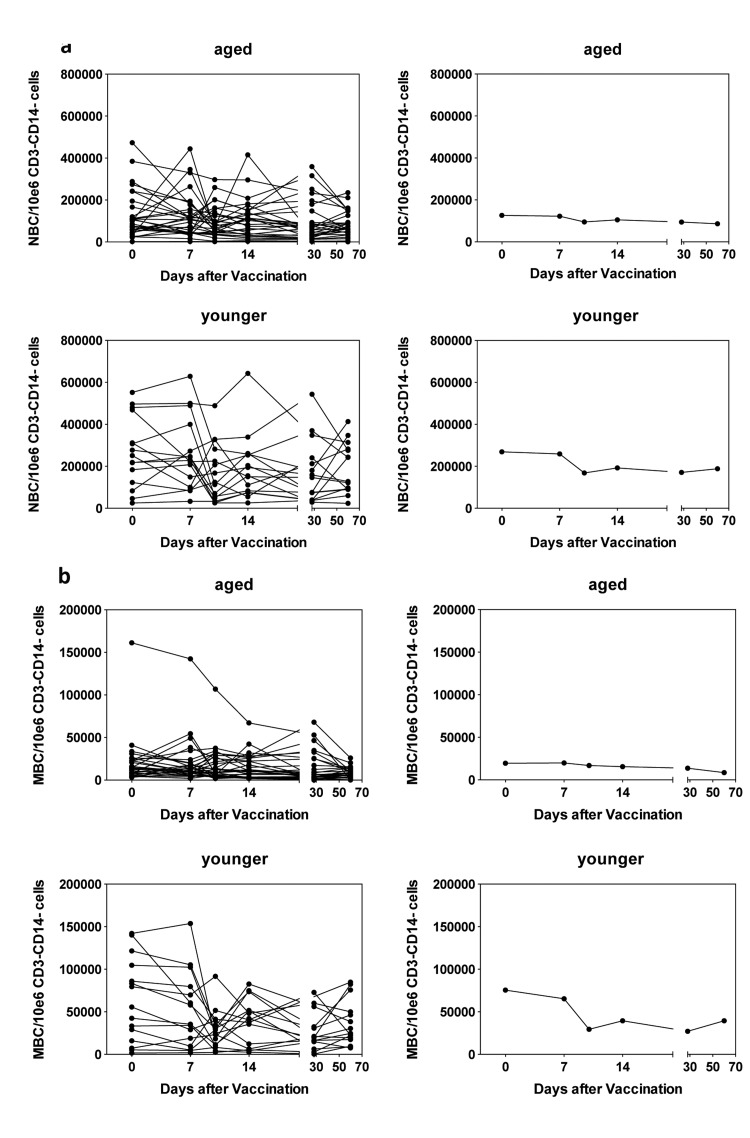
Circulating B cells **4a** shows numbers of naïve B cells per 10^6^ CD3^−^CD14^−^ live PBMCs. Naïve B cells (NBCs) were identified by gating on CD19^+^CD20^+^CD27^+^CD38^−^IgD^+^ cells. (**a**) shows data for PBMCs from individual aged subjects, (**b**) shows means of the same set of data, (**c**) shows data for PBMCs from individual younger subjects, (**d**) shows means for the same data. **4b** shows numbers of memory B cells per 10^6^ CD3^−^CD14^−^ live PBMCs. Memory B cells were identified by gating on CD19^+^CD20^+^CD27^+^CD38^−^IgD^−^ cells. The graphs are arranged as in 4A.

We compared numbers of the two major B cell subsets, i.e., naïve and memory B cells (Figure 4A,B) in young and aged individuals. Aged individuals had significantly lower number of naïve B cells at baseline (p = 0.004) and numbers remained stable after vaccination. In younger individuals, numbers significantly (p=0.045) declined by day 10 following vaccination and for the duration of the study remained below levels seen at baseline. Memory B cells were also higher in younger than aged individuals (p = 0.002) at baseline and in the both cohorts significantly declined by day 10 following vaccinations (p = 0.016 for the younger and 0.036 for the aged).

### Changes in Circulating ASCs

**Figure 5:**
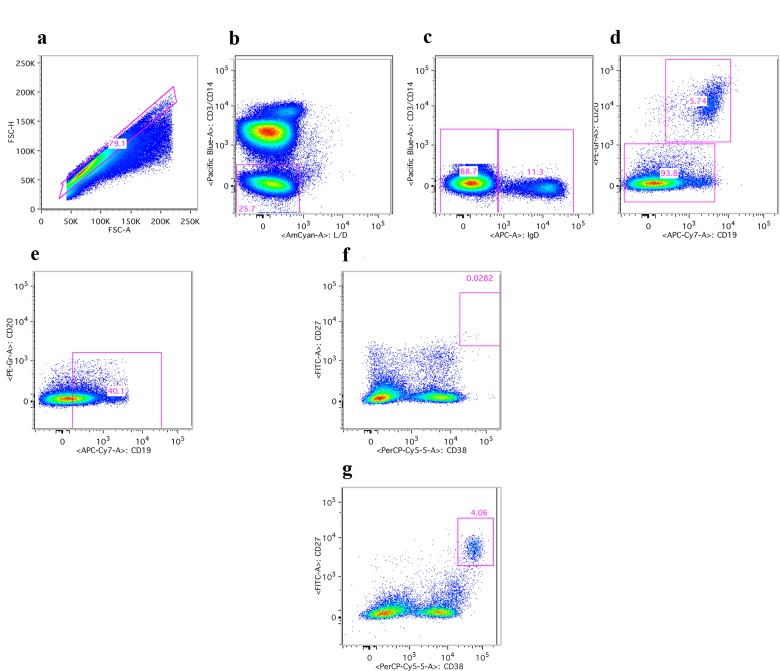
Gating scheme for ASCs Human PBMCs upon staining with antibodies were first gated on lymphoid cells (not shown). (**a**) shows gating on single cells. (**b**) shows gating on live CD3 and CD14 negative cells. (**c**) shows gating on IgD negative cells. (**d**) shows gating on CD20 negative cells. (**e**) shows gating on cells expressing intermediate to high levels of CD19. (**f**) and (**g**) show gating on ASCs, which are CD28 high and CD37 high. **F** shows results of a young individual at baseline; (**g**) shows the sample from the same individual harvested 7 days after vaccination.

**Figure 6:**
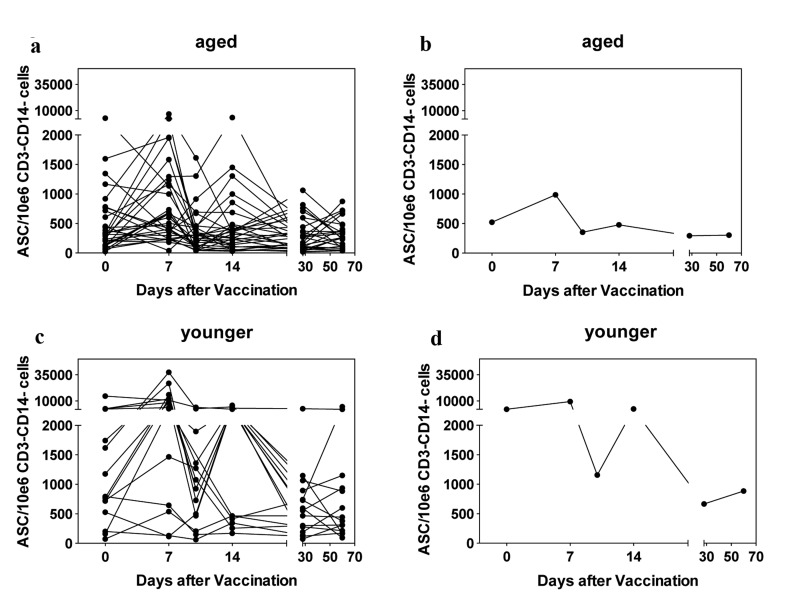
Circulating ASCs Graphs show numbers of circulating ASCs per 10^6^ CD3^−^CD14^−^ live PBMCs. Graphs are arranged as in Figure 4.

ASCs can be identified by cell surface markers that distinguish different B cell populations. Specifically, in humans ASCs are negative for IgD and CD20 but express high to intermediate levels of CD19 and high levels of CD38 and CD27. Upon influenza vaccination a transient rise in circulating ASCs is mainly reflective of vaccine-induced cells and up to 80% of such cells secrete antibodies specific to the antigens in the vaccine. PBMCs were therefore stained with lineage-defining antibodies before and at the indicated time points after vaccination. Samples were analyzed by flow cytometry and the post acquisition-gating scheme is shown in Figure 5, data are shown in Figure 6.

Individuals with more than 1000 circulating ASCs over 10^6^ CD3^−^CD14^−^live lymphocytes were defined as high baseline responders; subjects that showed an at least 2-fold increase in ASCs over baseline within 14 days following vaccination were deemed responders. Four out of 30 aged individuals and a significantly higher portion of 7 out of 15 young individuals (p = 0.04 by Fischer exact t-test) had high ASC counts at baseline. Younger individuals developed significantly higher numbers of ASCs upon vaccination than aged subjects (p = 0.013). Four younger subjects failed to show increases in ASCs within 2 weeks after vaccination, two of those had high ASC counts at baseline. Sixteen of the aged individuals failed to show a two-fold increase in ASCs and this included all of the subjects with high ASC numbers at baseline. Again correlation with antibody responsiveness to vaccination was poor, only one of the subjects that failed to show increases in ASC numbers failed to show an antibody response to the H3N2 virus of the vaccine.

Several human subjects reported symptoms indicative of an infection during the 60-day follow-up period of the study. Specifically 8 aged individuals reported coughs, runny noses or sinusitis at the 5^th^ or 6^th^ study visit by when vaccine-induced changes in ASC counts should have subsided. Of those 5 showed delayed increases in ASC counts, which coincided with their cold symptoms. Of the 6 younger individuals, who reported cold-symptoms at either of the last two visits, none developed accompanying increases in ASC counts.

**Figure 7:**
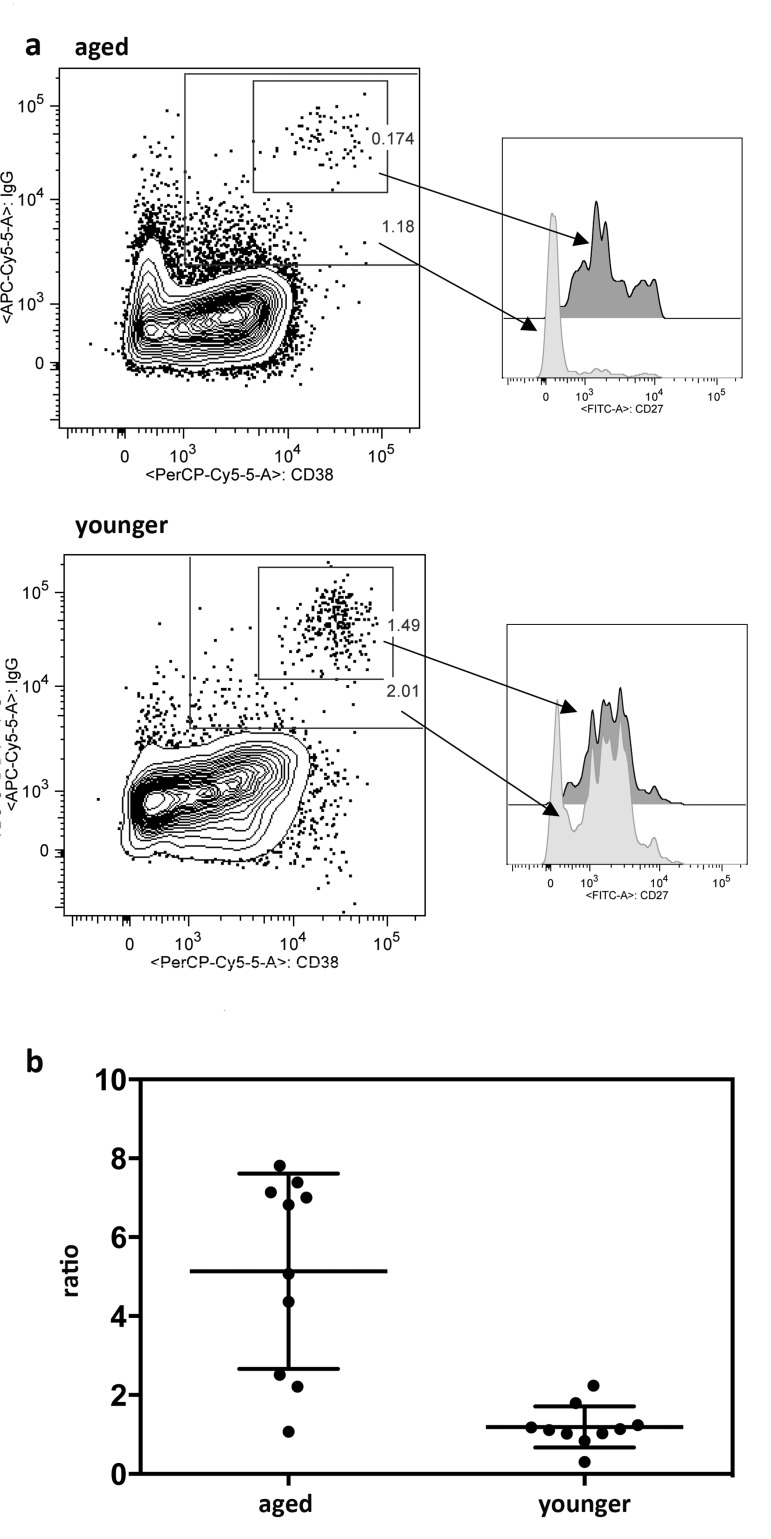
Expression of intracellular IgG In **7a** the graphs on the left show one representative example of PBMCs from one aged and one younger individual harvested 7 days after TIV vaccination. Cells were gated onto live CD3^−^CD14^−^IgD^−^CD20^−^CD19^+^ cells. They were then gated onto CD38 over IgG. The smaller gate shows CD38hiIgGhi cells, the larger gate shows cells that are CD38int-hi and IgG^int-hi^. Both gates were then as shown on the right gated onto CD27 over events. Dark histograms show CD27 expression on CD38^hi^IgG^hi^ cells, lighter histograms show CD27 expression on CD38^int-hi^IgG^int-hi^. **7b** shows the ratios of normalized cells obtained with the CD38^int-hi^IgG^int-hi^ gate as shown in (**a**) or the traditional ASC gate as shown in Figure 5. Graph shows results for day 7 post vaccination PBMCs of 10 individual aged and 10 younger subjects. Means and SDs are shown. cells.

Poor responses in the aged as measured by increases in circulating ASCs detected by high expression of CD38 and CD27 on CD20^−^IgD^−^CD19^+^ cells following vaccinations did not correspond to the results obtained by ELISpot assays, which revealed comparable influenza virus-specific ASC frequencies in blood of younger and aged individuals by day 7 following vaccination. This may have reflected that key markers, which were used for identification of ASCs by flow cytometry, were differentially expressed depending on age. We therefore repeated the cell stains with day 7 cryopreserved PBMC samples from 10 younger and 10 aged individuals including an intracellular stain for IgG, which is only present in isotype-switched ASCs. After flow cytometry, blots were gated on ASCs based on high expression of CD38 and CD27 as shown in Figure 4. Alternatively they were gated onto CD3^−^CD14^−^CD20^−^IgD^−^CD19^+^IgG^+^CD38^+^ cells relaxing the CD38 gate and including cells that showed intermediate expression (Figure 7A). In either cohort, cells that showed high expression of CD38 also carried high levels of IgG. A second population that was mainly detected in the aged expressed intermediate levels of CD38 and carried intermediate levels of IgG. Cells of this subset were largely CD27^−^. Of note CD20^+^ cells were IgG^−^, indicating that IgG^+^ cells that were detected by the analysis did not belong to the memory B cell pool, which express CD20 and surface IgG. Calculating numbers of ASCs based on either gating scheme and then comparing the results showed that in younger individuals, both gating schemes resulted in approximately equal numbers of ASCs indicating that cells positive for intracellular IgG were also high in CD38 and CD27. In the aged the latter gating scheme in most individuals resulted in markedly higher numbers of cells (p = 0.001) (Figure 7B) indicating that portion of the IgG producing cells in the aged had reduced expression of CD38 and lost expression of CD27.

### Relationship between Responsiveness to Vaccination and Previous Influenza Virus Exposures

**Figure 8:**
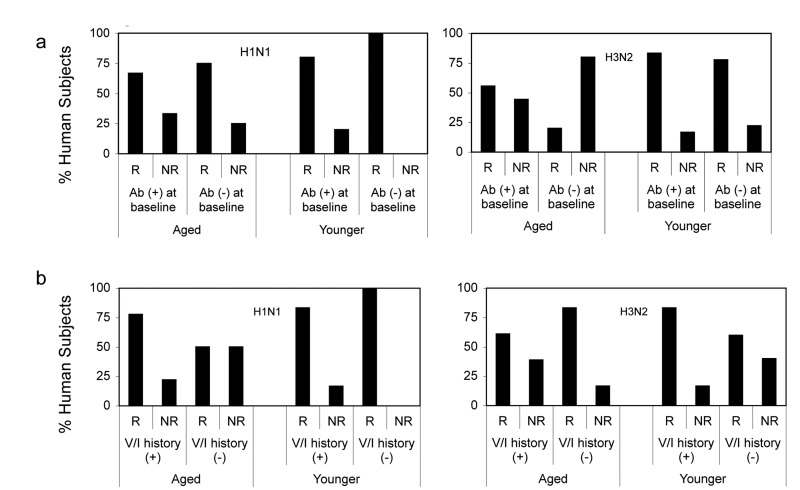
Responsiveness in Relation to Baseline Responses (**8a**) shows percentages of young and aged responders (R) defined as individual that showed 4 fold increases in neutralizing antibody titers following vaccination and non-responders (NR), which showed ≥ 4, fold increases. Ab (+) reflects individual with titers >1:40 at baseline, Ab (−) individuals had titers >1:40 at baseline. (**8b**) shows percentages of young and aged responders and non-responders in relation to their influenza vaccination (V) or infection (I) history. (+) indicates individuals who reported vaccinations or infections during the last 5 years; (−) indicates individuals that had neither been vaccinated nor infected during this time. Individuals that could not recall infections or vaccination during this period were excluded from this analysis.

To determine if pre-existing neutralizing antibodies affected responsiveness we analyzed responders versus non-responders according to baseline titers of or above 1:40 (sero-positive) or below 1:40 (sero-negative) (Figure 8A). Responses of both cohorts to either of the two vaccines were not significantly influenced by antibody titers at baseline.

**Figure 9:**
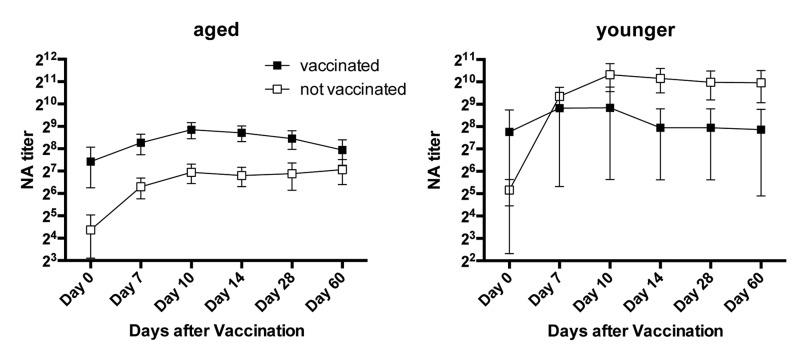
Responsiveness to H1N1 in Relation to vaccination in 2010 The graphs show average neutralizing antibody titers to H1N1 (±SEM) in aged and younger individuals that did or did not receive the influenza vaccine in 2010.

Vaccination history may influence vaccine-induced B cell stimulation. We therefore analyzed responders versus non-responders according to their history of influenza vaccinations or infections during the 5-year period prior to vaccination. For this analyses individuals that failed to report infections or vaccinations or were unsure were excluded. In the aged cohort, responders to H1N1 were more common in the subgroup that reported vaccinations or influenza-like illnesses while non-responsiveness was more common in individuals without recent exposure to influenza virus antigens. The only younger non-responder to H1N1 had been vaccinated in 2009. Aged responders to H3N2 more commonly had no recent exposures to influenza virus antigens than aged non-responders. In contrast 2 of the 3 younger non-responders to H3N2 virus did not recall recent influenza infections or vaccinations (Figure 8B). Although there were some trends based on vaccination history over the last 5-year period neither in the younger nor the aged did the effect of previous vaccination on responsiveness reach significance. We then narrowed the analyses to compare those that had been vaccinated in 2010, when the pandemic H1N1 virus was incorporated into the annual vaccines to those that did not receive the vaccine. Thirteen of aged and 3 of the younger cohort were vaccinated in 2010. In younger individuals there was no significant difference between those that did or did not receive the 2010 influenza vaccine although there was a trend of higher and more sustained responsiveness in those that did not. The aged that had been vaccinated in 2010 had higher neutralizing antibody titers to H1N1 at baseline although this did not reach statistical significance (p = 0.13). Upon TIV in 2011 the 2010 vaccine recipient developed higher H1N1 specific neutralizing antibody titers and this difference was significant on days 10 and 14 fter vaccination (Figure 9).

Lack of responsiveness by ELISpot assay to H1N1 was only seen in one individual without a recent history of infection or vaccination while one individual who failed to respond to H3N2 was unsure about infections or vaccinations within the last 5 years. All of the other aged non-responders reported to have received at least one dose of vaccine within this period. The one younger non-responder to H1N1 had been vaccinated, while of the 5 non-responders to H3N2, two had no history of recent vaccinations or infections.

Changes of ASC numbers of the aged were not influenced by recent influenza vaccinations or infections. Equal percentages of individuals that did or did not report recent vaccination or infection showed increases versus stable or decreasing ASC numbers following vaccination. In younger individuals only 50% of vaccinated individuals showed increases in ASC cells. In contrast the majority of younger subjects (80%) that failed to report recent vaccinations or infections developed markedly increased ASC responses. Nevertheless numbers of subjects were too low to determine significance of this difference.

## DISCUSSION

Here we tested acute B cell responses following TIV vaccination given in winter and spring of 2011/12 to cohorts of aged and middle-aged human subjects residing in the Triangle area of North Carolina. The study was undertaken to compare antibody and B cell responses of the two cohorts with regard to magnitude and kinetics of responses using three complementary assay systems.

A higher percentage of aged (63%) compared to younger individuals (27%) reported influenza vaccinations during the 5 years prior to the study. Nevertheless average neutralizing antibody titers and percentages of individuals with neutralizing antibody titer ≥1:40 at baseline to either of the two strains of influenza A viruses present in the vaccine were comparable between the two groups. In contrast binding antibodies of the IgG isotype to H1N1 and H3N2 were significantly higher prior to vaccination in the aged. This discrepancy may reflect that IgG antibodies in the aged at baseline were primarily directed to conserved non neutralizing epitopes that were shared between the vaccine strains and other previously circulating strains. Aged subjects that reported vaccinations or infections during the 5 years prior to this study had on average higher neutralizing and binding antibody responses at baseline as compared to individuals that had not been exposed to antigens of influenza virus. This difference reached significance for H3N2-specific neutralizing antibodies (p = 0.035) and for binding IgG to both viruses. Aged subjects that were first vaccinated after mid March of 2012 showed increased rates of neutralizing antibodies to the two influenza A virus strains prior to vaccination and differences in titers were significant for both viruses. In most years influenza activity is highest in January or early February and then declines. In the 2011/12-influenza season positive tests reported to the CDC did not peak till Mid March. It is thus tempting to speculate that an increased portion of aged subjects that were not vaccinated till Mid March had experienced subclinical infections, which had elicited B cell recall responses.

Upon vaccination most subjects of the younger age group showed increases in neutralizing antibody responses that were comparable between the two influenza A virus strains at all time points tested. Lack of neutralizing antibody responses was more common to the H3N2 virus and 2 out of the 3 younger non-responders to this virus were noteworthy since they lacked specific antibodies at baseline in contrast to the one younger non-responder to H1N1 who had robust baseline titers. Higher proportions of aged individuals failed to show increases in neutralizing antibodies to the H1N1 (27% of aged vs. 7% of middle-aged) or the H3N2 (33% vs. 20%) virus. After vaccination neutralizing antibody responses to H3N2 virus of aged individuals were comparable in magnitude to those of younger individuals. Peak antibody responses to H3N2 virus were seen at day 14 in the aged and were thus slightly delayed compared to those in younger individuals, which reached maximal responses by day 10. Antibody responses to H1N1 virus were compromised in the aged. Average titers upon vaccination, comparing titers either in all individuals or only in responding individuals were both significantly below those in younger individuals (p = 0.037 for all individuals, p = 0.011 excluding non responders) at all time points tested. These results are in contrast to a previous study, which reported increased antibody responses in the post pandemic phase to H1N1 vaccination [21] but in agreement with a study conducted in Singapore which also reported decreased responses to the H1N1 vaccine in aged individuals [22] and another study conducted prior to the 2009 pandemic which showed good responses to H3N2 and poor responses to H1N1 in the aged [20]. In our study aged subjects that reported neither influenza vaccinations nor infections during the 5 years prior to this study developed significantly lower titers to H1N1 upon vaccination (p = 0.011) as compared to those, who recalled either. Yet again, this is in contrast to a previous study, which showed that in adults between 22-49 years of age recent vaccination decreased antibody responses to vaccination with TIV [23] and may indicate that the aged are able to mount recall responses but have defective primary responses to TIV. Previous studies have reported that vaccine-induced antibody responses decline more rapidly in the aged [24]. In our study, although average titers in the aged declined more markedly by two months after vaccination than in younger individuals this difference was subtle and did not reach significance (p = 0.057).

Initially at day 7 after vaccination both cohorts showed significant increases in IgM to H3N2 and H1N1 suggestive of primary responses. Both cohorts also mounted significant IgG responses to both viruses, which are likely to reflect recall responses. Interestingly, the pattern of the IgG and to some degree the IgM responses differed between the younger and aged cohorts; IgG and IgM responses to H1N1 and IgG responses to H3N2 were higher on day 7 than day 28 in younger individuals while in the aged IgG and IgM titers to both viruses increased between days 7 and 28. The pandemic H1N1 virus was routinely incorporated into the annual vaccine as of 2010. Thirteen of the aged individual received the influenza vaccine in 2010. These individual had significantly higher levels of H1N1-specific IgG at baseline (p = 0.001). Upon vaccination their IgG responses to H1N1 were initially higher on day 7 than those of aged individuals that had not been vaccinated in 2010 but by day 28 after vaccination responses including isotypes of H1N1-specific antibodies were indistinguishable between the two aged subgroups. Neutralizing antibody titers became significantly higher in 2010 vaccine recipients on days 10 and 14 following vaccination in 2011. Again, this difference was not sustained.

Patterns of responsiveness assessed by the ELISpot assay, which detects all B cells producing antibodies to influenza virus regardless of their function, differed from those of circulating antibodies. Younger individuals more commonly had positive responses at baseline and were less likely to respond with 20% and 50% showing no responses to H1N1 and H3N2 virus respectively. Non-responsiveness in the younger cohort was with one exception only observed in subjects that had either ≥ 10 spots per 10^6^ PBMCs at baseline or had neutralizing antibody titers ≥1:40 at baseline; the one exception reported annual influenza vaccinations. Within the aged cohort only 11% failed to show increases of circulating B cells to H1N1 virus while 24% lacked responses to H3N2 virus. Lack of responsiveness by ELISpot in the aged was only seen in individuals with high numbers of spots at baseline or recent vaccinations.

In the younger cohort, peak ELISpot responses to both viruses were observed between days 7-14 with subjects that had high spot numbers at baseline typically responding later. The aged showed a dominant peak on day 7 followed in some individuals by a less pronounced increase on day 14. Two aged individuals showed delayed peaks on day 28 following vaccination, and both reported previous vaccinations and had high antibody titers at baseline. Magnitude of responses to either virus was not significantly different between the two cohorts.

The most pronounced age-related defects were seen upon staining of CD19^+^ PBMCs with antibodies to CD27 and CD38, which are typically used to identify ASCs. Upon exposure to antigen naïve and memory B cells are stimulated and after undergoing antigen-driven proliferation and/or hypermutations can transiently be detected in blood. It has been reported that, in young individuals by day 7 following influenza vaccination, up to 80% of circulating ASCs are specific to antigens of the vaccine [25]. Younger individuals had significantly higher baseline numbers of circulating ASCs as compared to the aged, as has been reported previously [26]. Upon vaccination 73% of the younger individuals showed marked increases in CD38^hi^CD27^hi^ ASCs by day 7, which then decreased by day 10 and again in 73% of individuals increased by day 14. Lack of increases on day 14 were seen in younger individuals that reported recent vaccination suggesting that the ASCs circulating on day 7 were primarily derived from memory B cells while those on day 14 reflected de novo stimulated B cells. Of the aged, only 37% vs. 30% showed increases in CD38^hi^CD27^hi^ ASCs by day 7 and 14 respectively.

Contradicting results obtained by two of the antigen-specific methods of antibody and B cell detection, i.e., the microneutralization and the ELISpot assays, with the former unlike the latter showing reduced responsiveness mainly to the H1N1 virus in the aged can easily be explained; the microneutralization assay only detects antibodies to neutralizing epitopes of the viral surface antigens while the ELISpot measures circulating ASCs to influenza virus regardless of their fine-specificity and thus allows for detection of B cells producing antibodies to non neutralizing and more conserved epitopes. Furthermore, influenza virus-specific ASCs of the aged may have produced on average lower numbers of antibody molecules, which would have resulted in reduced neutralizing antibody titers without affecting numbers of specific ASCs. We view this as unlikely as increases of H1N1-specific antibodies following TIV measured by ELISA were similar in aged and younger individuals, suggesting that aged B cells more commonly produced non neutralizing antibodies directed presumably to epitopes that are conserved between the 2011 H1N1 vaccine strain and previously circulating strains, a principle long known as “antigenic sin” [27]. Such cross-reactive antibodies may be less suited to provide protection against infection. Contradicting results obtained by surface staining and ELISpot assays, with the former indicating defective responses in the aged wile the latter showed comparable responses in both cohorts, are harder to reconcile. We, like others, identified ASCs by expression of CD19, a pan B cell lineage marker, and high expression of CD27 and CD38 on CD3^−^CD14^−^IgD^−^CD20^−^ cells. It is feasible that upon aging expression of these markers declines on ASCs. It is well known that in the elderly expression of the co-stimulator CD28 declines on T cells resulting in reduced T cell responsiveness [28]. CD27 is a member of the tumor necrosis family and signals through the tumor necrosis factor receptor-associated factors (TRAF)2 and 5 to activate nuclear factor kappa-light-chain-enhancer of activated B cells (NF-κB) and mitogen-activated protein kinase (MAPK)8 and JUN [29]. On B cells it is a marker of previous encounter with antigen and thus used to define memory B cells. CD27^−^ memory B cells have been described and it is thought that such B cells are either induced outside germinal centers [30]or prematurely leave germinal centers before they acquire CD27 [31]. CD27^−^ memory B cells show lower frequencies of somatic hyper-mutations compared to their CD27^+^ counterparts [32] suggesting either the lack of or incomplete maturation within germinal centers. In the aged numbers of circulating IgD^−^CD27^−^ memory B cells increase [33], which presumably reflects defects in germinal center formation and our results suggest loss of CD27 on recently activated circulating ASCs.CD38, which distinguishes ASCs from memory B cells, is also expressed on activated T cells and previous studies have shown a decline of CD8^+^CD38^+^ T cells during aging[34]. Cell surface expressed CD38 upon its ligation activates the phosphoinositide 3-kinase (PI3K) pathway, which through Protein Kinase B (Akt) and the mammalian target of rapamycin (mTOR) increases glucose uptake and glycolysis thus meeting the increased energetic needs of activated lymphocytes. Intracellular CD38, which was not assessed by our staining method, also cannibalizes nicotinamide adenine dinucleotide(NAD) [35] and thereby reduces the activity of sirtuin-1, a nuclear energy sensor which increases stress-resistance and influences metabolismthrough promotion of gluconeogenesis, fatty acid oxidation, and mitochondrial biogenesis [36,37]. Reduced surface expression of CD38 by aged ASCs would thus be expected to negatively affect cell metabolism, which is compatible with our finding of markedly lower levels of IgG in CD38^int^ as compared to CD38^hi^ cells.

In summary, in the 2011/12 influenza season aged individuals responded well to the H3N2 virus of TIV but mounted lower responses to the H1N1 virus. ASCs of the aged showed reduced expression of two signaling molecules which corresponded with reduced levels of IgG production, revealing a novel and hitherto undescribed defect of adaptive immunity that arises during immunosenescence.

## MATERIALS AND METHODS

### Viruses

Stocks of H1N1 A/California/04/2009 and H3N2 A/Perth/16/2009, the two influenza A virus strains of the 2011 influenza vaccines, were obtained from the Center for Disease Control, Atlanta, Georgia. Viruses were expanded in 10 day-old specific pathogen-free (SPF) embryonated eggs for 48hrs at 35°C. After 48hrs allantoic fluid from the infected eggs was isolated and concentrated by centrifuging at 20,000rpm for 1 hr at 4°C. The pellet was re-suspended in PBS and further purified by overlaying onto a 55-10% sucrose density gradient. The virus was titrated by hemagglutination assay with chicken red blood cells. The mean tissue culture infective dose (TCID_50_) was determined by serially diluting virus on Madin-Darby Canine Kidney (MDCK) cells and screening cells 3 days later for viral plaques. Infectious virus was used for neutralizing antibody assays or inactivated by a 45-minute treatment with betapropionolactone for ELISpot assays.

### Human Subjects

Blood was collected after informed consent from community dwelling persons in the Durham-Raleigh-Chapel Hill area of North Carolina. Younger individuals were 30-40 years of age; older individuals were ≥ 65 years of age. The following subjects were excluded from the study: [1] humans with immunosuppression resulting from diseases (e.g., clinically active malignancy, HIV/AIDS, immune disorders) or drugs (e.g., cancer chemotherapy, corticosteroid use); [2] individuals with significant underlying diseases that would be expected to prevent completion of the study; [3] subjects, which were bed-ridden or homebound or had intercurrent illnesses that might interfere with interpretation of study (e.g., urinary tract infection, respiratory tract infection); [4] individuals that were unlikely to adhere to protocol follow-up; [5] subjects that were involved in a conflicting study; [6] subjects that had a history of alcohol or substance abuse; [7] subjects with contraindication for influenza vaccination such as anaphylactic hypersensitivity to eggs or to other components of the influenza vaccine, and moderate or severe acute illness with or without fever, and Guillain-Barre Syndrome within 6 weeks following a previous dose of influenza vaccine. Persons with moderate to severe acute febrile illness were not vaccinated until their symptoms have abated.

From enrolled subjects demographic data and medical history including medical diagnoses, medications, vaccination to influenza and other infectious diseases, and history of influenza or influenza-like diseases during the last 5 years were recorded. Subjects were bled and then vaccinated with TIV via the intramuscular route in the deltoid muscle. Subjects were bled again and on days 7, 10, 14, 28 and 60 following TIV vaccination. Change in medical history, change in medications, influenza-like illness, adverse events (AE) and serious adverse events (SAE) were assessed by solicited reports at each study visit and unsolicited reports from subjects at any time after study enrollment. Solicited AEs included symptoms of influenza-like illness and injection site complaints, including pain, tenderness, redness, and swelling.

### Collection of Blood and Isolation of PBMCs and Plasma

Blood was collected into heparinized tubes and shipped overnight to Philadelphia. A 2 ml aliquot of each sample was set aside for serum collection. PBMCs were isolated from the remaining samples using established protocols. Specifically, blood was overlaid onto Ficoll-Paque Plus (GE Healthcare Biosciences, Piscataway Township, NJ) and spun for 30 minutes at 2000 rpm, with brake off, and at 50% acceleration. The PBMC layer at the Ficoll interface was then collected and washed twice with Hank's Balanced Salt Solution (Gibco, Grand Island, NY), by centrifuging at 2000 rpm. The washed, pelleted cells were then treated with 10 ml of red blood cell lysis buffer (eBioscience, San Diego, CA). Lysis was stopped by adding 5 ml of Roswell Park Memorial Institute (RPMI) medium supplemented with 10% fetal bovine serum [FBS] and washed using Hank's Salt (HBSS). Cells were then resuspended in 5ml of Dulbecco's modified Eagles medium (DMEM), live cells were counted using Trypan Blue as a diluent.

### Micro-Neutralization Assay

Influenza specific micro-neutralization assay was performed in 96 well plates. Briefly, heat-inactivated human sera were serially diluted (1:10 to 1:5120) in serum-free Minimal Essential medium (MEM) in a 96 well plate. Equal volume of the two Influenza strains, Influenza A/H1N1/2009/California and Influenza A/H3N2/2009/ Perth at 50TCID_50_ was added to serum samples and incubated for 1 hr at 37°C. After 1 hr, serum-virus mixtures were added to Madin Darby canine kidney (MDCK) cells and further incubated for 2 hrs at 37°C with 5% CO_2_. The plates were washed and replaced with MEM containing Tosyl phenylalanyl chloromethyl ketone-modified trypsin after incubation and scored for cytopathic effect after 3 days. The highest serum dilution in which 50% of the MDCK cells were intact was scored as the neutralization titer.

### ELISA

To measure H1N1/California and H3N2/Perth-specific antibody isotypes, wells of Nunc Maxisorp™ plate were coated with 10μg/ml of each virus in bicarbonate buffer overnight at 4°C. Isotype standards for IgA1, IgG and IgM (Athens Research & Technology, Inc., Georgia, USA) were also included in each plate. After coating, plates were washed and blocked with 3%BSA in PBS containing 0.05% Tween. Heat-inactivated sera of young and old subjects from day 0, 10 and 28 were diluted to 1/250 and added to the plate for 2h at room temperature, followed by washing 4X with PBST. Alkaline phosphatase conjugated mouse anti-human IgA1 at 1:1000, IgG at 1:3000 and IgM at1:1000 (SouthernBiotech, Alabama, USA) dilutions were added to the plates and incubated for 1h at room temperature. Plates were further washed 4X with PBST and developed using alkaline phosphatase substrate containing pNPP tablets (Sigma Aldrich, Missouri, USA) in DEA buffer and absorbance was recorded at 405nm. The absorbance values were plotted against standard curves from each plate for every isotype and the concentration was determined and is expressed in μg/ml.

### ELISpot Assay

96 well immobilin-P membrane plates (Millipore, Billerica, MA) were coated with 10μg/ml of H1N1 A/California/04/2009 or H3N2 A/Perth/16/2009 virus overnight. Negative control (PBS alone) and positive (total human Igs) wells were also included. The plates were washed 4X with PBS and blocked with 10% RPMI medium for 2hrs at 37°C. Freshly isolated PBMCs from human subjects were added onto the plate at 2×10^5^ cells/well in duplicates and incubated overnight in a humidified 5% CO_2_ incubator at 37°C. The plates were further washed 6X with PBS containing 0.05% Tween and incubated with alkaline phosphatase conjugated anti-human IgG (Sigma Aldrich, Missouri, USA) at 1:1000 dilution for 1 hrs at 37°C. After the incubation the plates were washed 6X with PBST and developed using alkaline phosphatase substrate kit (Vector Labs, Burlingame, CA) the spots were analyzed using CTL Immunospot (Cellular Technology, Ltd., Cleveland, OH). The spots/well was calculated by subtracting spots to negative control and normalizing spots to 10^6^ PBMCs cells.

### B cell Detection by Flow Cytometry

3 × 10^6^ cells of each sample were used for flow cytometric analyses. Cells were initially treated with Human TruStain FcX Fc Receptor Blocking solution (BioLegend, San Diego, CA) for 30 minutes, washed with PBS at 1500 rpm for 5 minutes and then stained with fluorochrome-conjugated antibodies. The following antibodies were used: CD19-APC-Cy7, CD20-PE (BioLegend), IgD-APC CD38-PerCPCy5.5, CD3-Pacific Blue, CD14-Pacific Blue, CD27-FITC, and AmCyan Aquablue as a live cell stain. The optimal concentrations of these antibodies were determined experimentally. Samples were stained for 30 minutes at room temperature, washed with PBS and then resuspended in 150μl of fixative (BD Pharmingen). All antibodies were obtained from BD Biosciences (San Jose, CA) unless specified differently. The stained samples were analyzed in a LSRII flow cytometer (BD Biosciences, San Jose, CA).

Cells were gated on lymphoid single cells and then on live cells that were negative for CD3 and CD14. For ASC identification IgD^+^ and CD20^+^ cells were excluded and CD19^hi^ cells were gated on CD38^hi^ and CD27^hi^. In some samples a stain for intracellular IgG was included. In these samples CD19^hi^CD38^+^ samples upon exclusions of cells expressing CD3, CD14, IgD or CD20 were gated onto IgG^+^ cells. Memory B cells were identified by gating on CD19^+^CD20^+^CD27^+^CD38^−^IgD^−^ cells. Naïve B cells were identified by gating onto CD19^+^CD20^+^CD27^+^CD38^−^IgD^+^ cells. To detect intracellular IgG, samples were stained for extracellular markers, as described above. The cells were then permeabilized using Cytofix/Cytoperm (BD Bio-sciences) for 30 minutes at 4°C. Cells were washed with Permwash (BD Biosciences) and stained with anti-human IgG-Alexa 700 (BD Biosciences) with Permwash as a diluent for 30 minutes. Samples were fixed, as described above.

### Statistical Analyses

Percentages of responders within the two cohorts were compared at baseline using Fisher's exact test. Baseline responses used t-tests, Mann-Whitney U-tests or two-part tests as appropriate. Continuous variables were log-transformed if needed to achieve normality. Repeated measures ANOVA were used to compare responses over time both between and within cohorts.
